# Isolation and characterization of a variant porcine epidemic diarrhea virus in China

**DOI:** 10.1186/1743-422X-9-195

**Published:** 2012-09-12

**Authors:** Yongfei Pan, Xiaoyan Tian, Wei Li, Qingfeng Zhou, Dongdong Wang, Yingzuo Bi, Feng Chen, Yanhua Song

**Affiliations:** 1 Guangdong Wen’s Group Academy, Guangdong Wen’s Foodstuffs Group Co, Ltd, Xinxing, Guangdong, 527400, China; 2 College of Animal Science, South China Agricultural University, Guangzhou, 510642, China

**Keywords:** Porcine epidemic diarrhea virus, Virus isolate, Variant

## Abstract

An outbreak of diarrhea in pigs started in Guangdong, South China in January 2011. Cases were characterized by watery diarrhea, dehydration and vomiting, with 80–100% morbidity and 50–90% mortality in suckling piglets. The causative agent of the diarrhea was ultimately identified as porcine epidemic diarrhea virus (PEDV). In this study, we isolated a PEDV strain designated CHGD-01 from piglet intestines using Vero cell cultures, and its specific cytopathic effects were confirmed in susceptible cells by direct immunofluorescence testing and electron microscopy. The complete genome of CHGD-01 was shown to be 28,035 nucleotides in length, with a similar structure to that of PEDV reference strains. Phylogenetic analyses based on the whole genome revealed that CHGD-01 shared nucleotide sequence identities of 98.2–98.4% with two other Chinese isolates reported in the same year, thus constituting a new cluster. Amino acid sequence analysis based on individual virus genes indicated a close relationship between the spike protein gene of CHGD-01 and the field strain KNU0802 in Korea. Its ORF3 and nucleoprotein genes, however, were divergent from all other sequenced PEDV isolate clusters and therefore formed a new group, suggesting a new variant PEDV isolate in China. Further studies will be required to determine the immunogenicity and pathogenicity of this new variant.

## Background

Porcine epidemic diarrhea virus (PEDV) is the causative agent of porcine epidemic diarrhea (PED), an enteric disease characterized by vomiting, watery diarrhea, and dehydration in swine. This disease was first reported in feeder and grower pigs in the UK in 1971 [1], after which the virus was identified [2,3]. The disease has subsequently been reported in a number of European countries [4,5], and more recently in China, Korea, Japan, Thailand and Vietnam [6-12].

PEDV is an enveloped RNA virus belonging to Group 1a, genus *Coronavirus*, family *Coronaviridae*, within the order *Nidovirales.* The viral genome is a single-stranded positive-sense RNA of approximately 28 kb in size, containing six genes: the replicase (Rep), spike (S), ORF3, envelope (E), membrane (M), and nucleoprotein (N) genes, arranged in the order 5’-Rep-S-ORF3-E-M-N-3’ [13-15]. As a coronavirus, PEDV comprises three corresponding major viral structural proteins: the S (180–220 kDa), M (27–32 kDa), and N (55–58 kDa) proteins [16,17]. The S protein plays a pivotal role in determining viral-cellular fusion activity and in inducing an immune response in the natural host [18-20]. The M protein plays an important role in the virus-assembly process, and induces antibodies that neutralize virus in the presence of complement [21-23]. The N protein of coronaviruses forms a helical ribonucleoprotein with the virus genomic RNA and is the predominant antigen produced in coronavirus-infected cells, thus making it a major viral target [24,25]. Unlike the structural proteins, little is known about the functions of the accessory proteins. The recently-identified ORF3 gene has been demonstrated to be a potentially important determinant of virulence in this virus [26,27].

PEDV can generally be controlled using the vaccine strategy, and vaccination with killed or attenuated PEDV vaccine has been widely carried out in China, where PED usually manifests a mild and enzootic pattern (lower mortality in suckling piglets). However, a severe acute diarrhea outbreak associated with high morbidity (80–100%) and mortality (50–90%) was observed in suckling piglets at approximately 10 premises in Guangdong, China, in early 2011. Although most sow herds had previously been vaccinated with both killed and attenuated PEDV vaccines based on CV777, some of these were still infected, showing transient diarrhea and anorexia, but not death. PEDV field isolates thus need to be isolated and their molecular epidemiology investigated in order to better control and prevent future PEDV outbreaks. In this study, a PEDV strain was isolated from sick piglets during this outbreak and grown in Vero cells. Molecular characterization of the virus identified it as a variant PEDV emerging in China.

## Results

### Pathogen detection

For PCR/RT-PCR detection of viruses, intestinal and fecal samples from 33 sick piglets were first examined for the presence of TGEV, porcine rotavirus, porcine reproductive and respiratory syndrome virus (PRRSV), PCV2, porcine kobuvirus and PBoV. All assays were negative except for three PCV2-positive samples. All specimens were subsequently examined for PEDV, and all samples were positive (data not shown).

All samples were tested for common pathogenic intestinal bacteria, such as *Salmonella* and pathogenic *Escherichia coli*, but no significant numbers of *Salmonella* or *E. coli* were isolated from sick piglets from different premises.

### Virus isolation and identification

**Figure 1:**
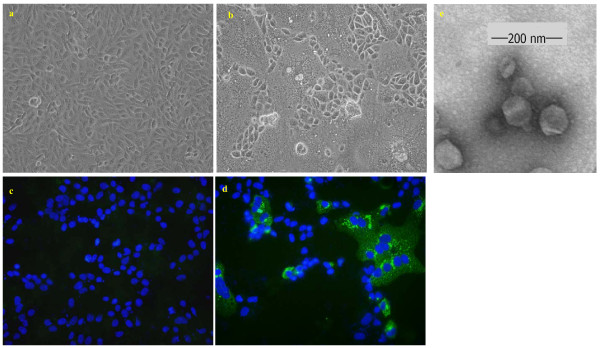
**Identification of CHGD-01 isolate by optical microscopy, IFA assay and electron microscopy.** (**a**) Mock-infected Vero cells. (**b**) CPE in Vero cells infected with PEDV-CHGD-01 showing syncytia and multiple nuclei. (**c**) IFA in non-infected Vero cells. (**d**) IFA in CHGD-01 isolate-infected Vero cells. Original magnification × 200. (**e**) Morphology of CHGD-01 isolate under electron microscopy (negatively stained); bar = 200 nm.

A distinct CPE was noted after seven passages in Vero cells. The CPE was characterized by cell fusion, syncytia formation and eventual detachment from the plastic surface (Figure 1b). The virus isolate designated CHGD-01 was biologically cloned by three rounds of plaque purification in Vero cells prior to further virus characterization. In addition, cells tested positive by IFA (Figure 1d). Electron microscopy of a negatively-stained sample revealed the presence of medium-sized viral particles of approximately 80–120 nm in diameter. In some of the virions, surface projections characteristic of coronaviruses were evident (Figure 1e).

### Sequencing results and phylogenetic analyses

A total of 28,035 nucleotides were determined for CHGD-01, encompassing the Rep, S, ORF3, E, M, and N proteins. Alignment of the genome sequence with those of TGEV, human coronavirus-229E and PEDV reference strains available in GenBank showed the highest identity with Chinese PEDV BJ2011-1 (98.4%), which was isolated in 2011. Phylogenetic analysis showed that CHGD-01 formed a new cluster together with BJ-2011-1 and CH/FJND-3/2011.

**Figure 2:**
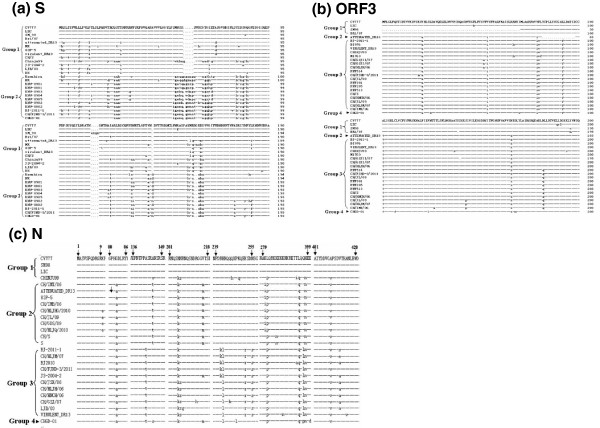
Multiple alignments of the S (a), ORF3 (b) and N (c) amino acid sequences of PEDV identified in China and other countries.

**Figure 3:**
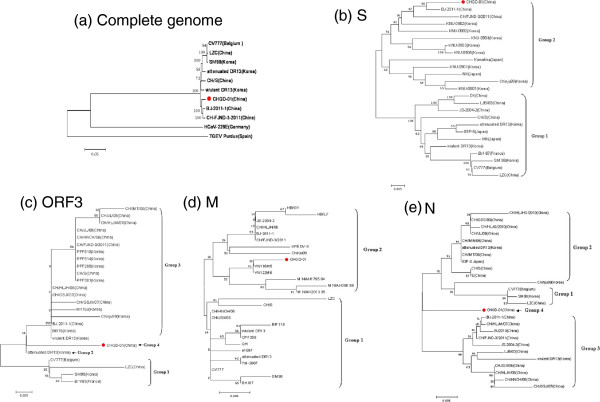
**Phylogenetic analysis by neighbor-joining method (bootstrapping for 1000 replicates with a value >70%) based on nucleotide sequences of full-length genomes (a) and amino acid sequences of different proteins (b: S; c: ORF3; d: M; e: N).** Sequence of CHGD-01 strain is indicated by the filled circle.

The S protein of CHGD-01 was 4158 nucleotides long, encoding a protein of 1385 amino acids. Compared to the PEDV reference strain CV777, two insertions (in positions 61–64 and 136) and one deletion (in position 158–159) were observed (Figure 2a). It shared 92.6–98.2% amino acid identity with other PEDV strains, and the highest identity with the Chinese strain BJ-2011-1. Phylogenetic analyses of the S protein amino acid sequences revealed that all PEDV strains in this study could be separated into two groups: CHGD-01 belonged to Group 2, which also contained the two Japanese isolates Kawahira and NK, eight Korean field strains (Chinju99, KNU-0801, KNU-0802, and KNU-0901–KNU-0905) and two Chinese strains (BJ-2011-1 and CH/FJND-3/2011), which were deposited in GenBank in 2011 (Figure 3b).

The ORF3 gene of CHGD-01 was determined to be 675 nucleotides in length, coding for a polypeptide of 225 amino acids. The ORF3 gene of CHGD-01 shared 93.3–97.3% amino acid identity with other PEDV strains, and the highest identity with the Korean virulent DR13. Based on phylogenetic analyses of the ORF3 amino acid sequences, all the PEDV strains could be divided into four groups (Figure 3c). Group 1 comprised CV777, Br1/87, SM98 and LZC strains; Group 2 consisted of vaccine strains (attenuated DR13); Group 3 was made up of eight Korean field strains and 11 Chinese strains. These results showed some similarities with the previous report by Park et al. [28]. Interestingly, phylogenetic analysis showed that CHGD-01 formed a fourth group, clearly different from other PEDV isolates. Each group had unique differences in its sequences. Group 2 had a 17-amino acid deletion (at positions 82–98), which is a marker of attenuated vaccine. ^F^80^V^ occurred only in Group 3, while ^L^92^F^ existed in Groups 3 and 4. Groups 2, 3 and 4 shared five specific amino acid changes (^V^21^A^, ^V^54^I^, ^V^78^I^, ^F^79^V^, ^A^101^T^ and ^N^166^S^). An additional five unique mutations (^L^25^S^, ^I^70^V^, ^C^107^F^, ^F^124^L^ and ^D^168^N^) occurred in CHGD-01 (Figure 2b). These mutations might play an important role in the classification of the groups.

The M protein of CHGD-01 was found to be 226 amino acids long and highly conserved. It shared 96.9–99.5% amino acid identity with the other PEDV strains. The highest amino acid identity (99.5%) was with the Vietnamese field isolates (VN116M5 and VN116M6), which were isolated from southern Vietnam during the 2009–2010 PED outbreaks. Phylogenetic analyses demonstrated that all PEDV strains were similarly divided into two distinct genetic groups (Figure 3d). Group 1 comprised six Chinese strains (LZC, CH/S, CH/HNCH/06, CH/JSX/06, QH, YM-2007), seven Korean strains (BIF118, CPF259, e1697, SM98, DR13 and its attenuated counterpart) and two European strains (CV777 and Brl/87). The CHGD-01 strain belonged to Group 2, which comprised seven Chinese strains (HB/GY, HB/LF, JS-2004-2, BJ-2011-1, CH/FJND-3/2011, CHGD-01), two Korean strains (KPEDV-9 and Chinju99), the recent Vietnamese strains (VN166M5 and VN122M6) and Thailand strains (NIAH1795, NIAH380 and NIAH2013). Previous studies suggested that JS-2004-2 was the ancestor of the Vietnamese and Thailand PEDV strains, and our results also demonstrated a close relationship between CHGD-01 and JS-2004-2.

The N gene of CHGD-01 was 1326 nucleotides in length, coding for a polypeptide of 441 amino acids. The amino acid sequence had 95.9–97.7% identity with other PEDV strains, and the highest identity (97.7%) with the CH/S and vaccine strains (attenuated DR13). Alignment analysis indicated that the entire nucleocapsid protein of CHGD-01 was generally highly conserved, but had 16 amino acid substitutions compared to CV777. It was predicted to contain eight potential serine (S)-linked phosphorylation sites and six potential threonine (T)-linked phosphorylation sites, including two protein kinase C phosphorylation sites, one casein kinase II phosphorylation site, and one cAMP- and three cGMP-dependent protein kinase phosphorylation sites. Based on analyses of the amino acid sequences of the PEDV N proteins, all PEDV strains could be divided into four groups (Figure 3e). CHGD-01 differed from all other PEDV isolates, and formed a new separate group. There were unique changes in the deduced amino acid sequences among the groups. Two amino acid changes (^A^145^T^, ^V^216^M^) were found in Group 2; ^K^252^R^, ^N^255^S^ and ^Q^397^L^ occurred only in Group 3; Groups 3, 4 shared two specific amino acid changes (^S^142^T^, ^H^242^L^); and ^G^84^A^ was found in Groups 2, 3 and 4. Noticeably, six amino acid mutations (^A^145^S^, ^N^157^S^, ^K^248^L^,^A^293^T^, ^I^322^V^, ^Q^397^P^) were observed only in CHGD-01 (Figure 2c).

## Discussion

The clinical signs from the outbreak in Guangdong, along with both macroscopic and microscopic examinations of the lesions, were strongly suggestive of PEDV or TGEV infection. TGEV and other possible pathogens were excluded and PEDV was therefore focused on as the only significant causative agent, followed by attempts to isolate it from piglets with the disease. PEDV was isolated successfully and propagated in Vero cell cultures in the presence of trypsin, according to the method described by Hofmann and Wyler, and by Kusanagi et al. [29,30]. However, the CPE caused by the PEDV isolate was not detected in Vero cells until after seven passages, despite being detectable by PCR. This was in contrast to the results of Hofmann and Wyler, who observed a CPE even at passage one in the same cells, and a prominent effect from virus passage five [29]. Kusanagi et al. reported that a characteristic CPE was detected at passage two, and became more evident with subsequent passages of Vero cells [30]. This discrepancy may be attributable to the relative susceptibilities of Vero cells to different isolates of PEDV.

To characterize the virus isolate, we determined the complete genomic sequence of CHGD-01 and analyzed the phylogenetic relationships among PEDV strains at the genomic and individual gene levels. The most variable regions were located in the S and ORF3 genes. The S protein is involved in receptor binding and virus entry, the induction of neutralizing antibodies, and host-cell fusion [18-20]. It is also an important target for monitoring the genetic diversity of coronavirus isolates [31,32]. Most PEDV reference isolates prior to 2007, including all Chinese strains, were partitioned into the first cluster (Group1; G1), whereas the three Chinese strains (CHGD-01, BJ-2011-1 and CH/FJND-3/2011) belong to the second cluster (Group 2; G2). All these three strains were isolated in 2011 when severe PED outbreaks were rampant in some areas of China. All Korean field strains belong to this cluster (except the Japanese isolates Kawahira and NK), most of which were isolated in 2008 and 2009. Interestingly, these three Chinese strains formed a unique cluster with the highest amino acid identities to KNU-0802 (96.5%, 96.9% and 96.2%, respectively). In this study, the same amino acids insertions and deletion of the S gene were observed among three Chinese (CHGD-01, BJ-2011-1 and CH/FJND-3/2011) and two Korean (KNU-0802 and KNU-0902) strains. Both KNU-0802 and KNU-0902 were isolated in South Korea during 2008–2009. These results suggest that the recently isolated Chinese strains may have originated from Korean ones.

The ORF3 gene is situated between the S and E genes in the PEDV genome, but its function is unknown. Previous studies found that a continuous 49 or 51 nucleotide region was deleted within the ORF3 gene when PEDV was repeatedly passaged in cell culture, indicating a possible involvement in viral pathogenicity [33,34]. Recent investigations demonstrated that all the reported PEDV isolates could be classified into three groups, based on phylogenetic analyses of the ORF3 genes [28,34]. Our study notably revealed that the amino acid sequence of ORF3 in the CHGD-01 strain was clearly different from those of all other PEDV isolates. This difference could not be attributed to the effects of limited cellular passaging, because the ORF3 gene sequence of CHGD-01 passage 0 cells (i.e. intestinal fecal sample) was almost identical to that of passage 10 cells (data not shown). CHGD-01 fell out with the three previously identified groups, and thus constitutes a new group. The CHGD-01 isolate showed 93.3–95.1%, 97.6%, and 96.0–97.3% amino acid sequence identities with members of Groups 1, 2 and 3, respectively. Genomic recombination is known to occur at high frequency between heterogenous genomes of coronaviruses. However, recombination analysis provided no evidence to suggest that CHGD-01 was derived directly from the recombination of known PEDV strains. Further molecular epidemiological evidence is needed to determine the origin and evolution of the CHGD-01 genome. The variations within the CHGD-01 genome, especially in the ORF3 and S genes, place it in a new cluster with BJ-2011-1 and CH/FJND-3/2011, based on phylogenetic analysis of the PEDV genomes.

PED was generally considered to be under control or had only mild effects in swine herds in China before the beginning of 2011. Since then, however, PED has unexpectedly devastated many swine farms, including those were killed and attenuated vaccines based on CV777 were being used, suggesting that these vaccines were no longer able to confer protection. This raised the question of whether any obvious changes in antigenicity and/or virulence are associated with the genetic variations demonstrated in CHGD-01. The results of this study suggest that there is an urgent need for more fundamental research aimed at understanding the basic biology of this virus strain, as well as the mechanisms of immunogenicity and pathogenesis of PEDV.

## Conclusion

The present study isolated and identified a PEDV CHGD-01 strain from infected piglets in Guangdong in 2011. Analysis of the genetics and evolution of CHGD-01 demonstrated significant genetic diversity compared to other PEDV reference strains, suggesting the presence of a new variant PEDV in China.

## Materials and methods

### Case description

Outbreaks of diarrhea were observed at a number of pig farms at different locations between January and March 2011. The cases were characterized by watery diarrhea, dehydration and vomiting, with 80–100% morbidity and 50–90% mortality in suckling piglets, whereas affected sows were characterized by diarrhea, anorexia, and depression, but recovered within 1 week. Breeding herds had been immunized with attenuated or killed PED-TGE combined vaccine produced in China in the fall and winter of the previous year. The PEDV component of this vaccine was based on the CV777 strain.

Sick or dead piglets aged 1–21 days were submitted for laboratory investigation. Necropsy examination of all piglets revealed that the small intestines were congested and filled with fluid, and were thin-walled as a result of severe mucosal atrophy. Microscopically, marked cytoplasmic vacuolation and exfoliation of enterocytes with subsequent shortening of villi were noted. Tissues and feces and/or blood were collected from live or dead animals submitted for polymerase chain reaction (PCR) detection to perform a surveillance.

### Screening for viral pathogens

Samples from feces and intestine tissues were subjected to virological investigations for common viral swine pathogens such as PEDV, transmissible gastroenteritis virus (TGEV), porcine rotavirus (PRV), porcine circovirus 2 (PCV2) [35], porcine kobuvirus [36], and porcine bocavirus (PBoV) [37], using previously described methods.

### Virus isolation and identification

Growth medium (GM) was Dulbecco’s modified Eagle’s medium (DMEM, Gibco, USA) supplemented with 10% heat-inactivated fetal calf serum, 0.3% tryptose phosphate broth (TPB), and antibiotics. Maintenance medium (MM) consisted of DMEM supplemented with 0.3% TPB and 10 μg/ml trypsin (Gibco). Virus isolation was performed using Vero cells (CCL-81™, ATCC), as described previously [29,30] with minor modifications. Briefly, intestinal samples positive for PEDV by reverse transcription (RT)-PCR were further filtered through a 0.22-μm syringe filter (Millipore, USA) and used as inoculum. GM was removed from confluent monolayer cell cultures, which were then washed twice with DMEM and inoculated with the filtered intestinal content suspensions. After adsorption for 60 min at 37 °C, the cells were washed with DMEM and MM was added. The Vero cell cultures were observed for 5 days for cytopathic effects (CPE).

Immunofluorescence assay (IFA) and electron microscopy were used to detect PEDV in the infected cells. The IFA utilized a 1:1000 dilution of mouse anti-S monoclonal antibody (Cat no: 9191, JBT, Korea) specific for PEDV and a 1:100 dilution of fluorescein isothiocyanate-conjugated goat anti-mouse IgG (Cat no: 02-18-06, KPL, USA). For electron microscopy, infected cell culture supernatants were partially purified by ultracentrifugation through a 20% (wt/wt) sucrose cushion, negatively stained with 2% ammonium molybdate, and examined with an electron microscope (JEM-1400, JEOL Ltd., Japan).

### Sequencing and sequence analysis

Viral RNAs were extracted from CHGD-01-infected Vero culture supernatant using TRIzol LS reagent (Invitrogen, USA), according to the manufacturer’s instructions. Twelve pairs of oligonucleotide primers were used to amplify the different regions of the CHGD-01 genomes, and were designed based on the sequences of PEDV strain CV777. The PCR products were purified and cloned into pMD18-T vector (TaKaRa, Japan) and sequenced using an automated genome sequence (Genetic Analyzer 3730XL; Applied Biosystems, USA). The terminal sequences were acquired using a kit for rapid amplification of cDNA ends (RACE) (Clontech, Japan). All primers are listed in Table 1. Sequence data were assembled and analyzed using Lasergene software (DNAstar Inc., USA). Multiple sequence alignments were performed using Clustal X 2.1 [38]. Phylogenetic analyses were carried out using the MEGA 4 program [39]. Phylogenetic trees based on the amino acid sequences of the S, ORF3, M and N proteins were elaborated using the neighbor-joining method, with bootstrapping over 1,000 replicates. The PEDV strains utilized in the present study including complete genome, S, ORF3, M and N genes are listed in Table 2, along with their GenBank accession numbers. Protein kinase-specific phosphorylation sites were identified using the prediction tool KinasePhos program through the web server (http://kinasephos.mbc.nctu.edu.tw/predict.php) [40]. The gene sequence was scanned for possible recombination events using the software package SimPlot (v 3.5.1), according to the methods described previously [41]. The genome sequence of CHGD-01 was registered in GenBank under the accession number JX261936.

**Table 1:** Primers used for sequencing reactions

**Oligonucleotides**	**Location**^**a**^	**sequence**
1 F	190-209	GCGTTCCGTCGCCTTCTACA
1R	2751-2729	CAGGAATCTGGAAGACACTTGCA
2 F	2663-2684	GTATTATGCCACCAGTGTCCCA
2R	4957-4938	CAGTTGCCAGCAGGCACTGT
3 F	4887-4906	ACCAGCGGTGCATTGCTTGA
3R	7475-7453	CAATGTGCTCTTGCAATCCTGCA
4 F	7327-7350	CTGTTAAGTTAGTGGACTCAGCGT
4R	9875-9856	ACTAGCGCCTTCAACTTGCA
5 F	9712-9731	GCGCTTGTGGTTCACCTGGT
5R	12259-12240	GGATCCACAGCGAAAGCGCA
6 F	12182-12202	ACGCTTGCAGGCTGGTAAACA
6R	14462-14442	TGGGCAGTGCTCTATCGCACT
7 F	14322-14341	ATACTAGGGGCGCTTCGGTT
7R	16780-16760	GTCAGGGTGCACAGGAATGAA
8 F	16662-16684	GTATGTGTGCCCTTAAGCCTGAT
8R	19002-18980	GTAAGTGGACGTTCGGCTTCATA
9 F	18874-18898	CGTAGCTTTTGAGTTGTATGCCA
9R	21330-21309	GCAATTAGCTGTACAGGGTTCA
10 F	21080-21101	CCATTCCAGCTTATATGCGTGA
10R	23487-23465	GTACATGTGAAGCTTCTCAGCGT
11 F	23272-23292	GTGTACGATCCTGCAAGTGGC
11R	25715-25694	TCACCTCATCAACGGGAATAGA
12 F	25535-25557	TCGTCCAATTGGTTAATCTGTGC
12R	27840-27820	TACCGTTGTGTGCAAGACCAA
5’ RACE Primer	307-288	TTGCTAGCCATAGCCGGCAG
3’Race Primer	27725-27747	CTATGTTCCAGGGTAGTGCCATT

^a^Location corresponds to position within the CV777(AF353511) genome.

**Table 2:** PEDV strains investigated in this study

**Strain/Isolate**	**Accession No, Country**	**Strain/Isolate**	**Accession No, Country**
***(a) Complete genome***		VN122M6	HQ883484, Vietnam
TGEV Purdue	AJ271965, Spain	Chinju99	DQ845249, Korea
HCoV-229E	AF304460, Germany	KPEDV-9	AF015888, Korea
CV777	AF353511, Belgium	e1697	FJ687454, Korea
SM98	GU937797, Korea	BIF118	FJ687460, Korea
attenuated DR13	JQ023162,Korea	CPF259	FJ687465, Korea
Virulent DR13	JQ023161, Korea	***(d) N gene***	
LZC	EF185992, China	LJB/03	DQ072726, China
CH/S	JN547228, China	JS-2004-2	AY653206, China
CH/FJND-3/2011	JQ282909, China	S	DQ355223, China
BJ-2011-1	JN825712, China	CH/IMB/06	FJ473387, China
CHGD-01	JX261936, China	CH/JSX/06	FJ473388, China
***(b) S gene***		CH/HNCH/06	FJ473389, China
Br1-87	Z25483, France	CH/HLJH/06	FJ473390, China
NK	AB548623, Japan	CH/IMT/06	FJ473391, China
MK	AB548624, Japan	CH/HLJM/07	FJ473393, China
83P-5	AB548618, Japan	CH/GSJ/07	HM210880, China
Kawahira	AB548622, Japan	CH/JL/09	HM210881, China
Chinju99	AY167585, Korea	CH/GDS/09	HM210882, China
KNU-0801	GU180142, Korea	CH/HLJQ/2010	HQ455345, China
KNU-0802	GU180143, Korea	CH/HLJHG/2010	HQ455346, China
KNU-0901	GU180144, Korea	BJ2010	JF690780, China
KNU-0902	GU180145, Korea	Chinju99	AF237764, Korea
KNU-0903	GU180146, Korea	83P-5	AB618615, Japan
KNU-0904	GU180147, Korea	***(e) ORF3 gene***	
JS-2004-2	AY653204, China	Br1/87	Z24733, France
DX	EU031893,China	CH/HNCH/06	GU372738, China
LJB-03	DQ985739, China	CH/HLJH/06	GU372732, China
***(c) M gene***		CH/IMT/06	GU372739, China
Br1/87	Z24733, France	CH/GSJI/07	GU372737, China
JS-2004-2	AY653205, China	CH/GSJII/07	GU372742, China
CH/HLJH/06	EU033964, China	CH/HLJM/07	GU372735, China
CH/HNCH/06	EU033963, China	CH/JL/08	GU372734, China
CH/JSX/06	EU033967, China	CH/JL/09	GU372741, China
YM-2007	EU302820, China	Chinju99	EU792474, Korea
QH	AY974335, China	PFF285	HQ537449, Korea
HB/GY	JN400910, China	PFF381	HQ537451, Korea
HB/LF	JN400909, China	PFF513	HQ537452, Korea
M_NIAH380_98	EU581712, Thailand	PFF514	HQ537453, Korea
M_NIAH1795_04	EU542415, Thailand	M1763	HQ537438, Korea
M_NIAH2013_95	EU581711, Thailand	BI976	HQ537433, Korea
VN116 M5	HQ883483, Vietnam		

## Abbreviations

PEDV: porcine epidemic diarrhea virus; TGEV: porcine transmissible gastroenteritis virus; CPE: cytopathic effects; RT-PCR: reverse transcription-polymerase chain reaction; RNA: ribonucleic acid; DMEM: Dulbecco’s modified Eagle’s medium; TPB: tryptose phosphate broth; GM: growth medium; MM: maintenance medium; IFA: immunofluorescence assay.

## Competing interests

The authors declare that they have no competing interest.

## Authors’ contributions

YFP, XYT, WL carried out most of the experiments and drafted the manuscript. FC, YHS critically revised the manuscript and the experimental design. QFZ, DDW and YZB contributed to the interpretation of the findings and revised the manuscript. All of the authors read and approved the final manuscript.
